# Amelioration of liver fibrosis with autologous macrophages induced by IL-34-based condition

**DOI:** 10.1186/s41232-025-00364-7

**Published:** 2025-01-24

**Authors:** Yuichi Igarashi, Haruka Wada, Masato Muto, Ryohei Sone, Yoshinori Hasegawa, Ken-ichiro Seino

**Affiliations:** 1 Division of Immunobiology, Institute for Genetic Medicine, Hokkaido University, Sapporo, Japan; 2 MEDINET Medical Institute, MEDINET Co., Ltd., Tokyo, Japan; 3 Laboratory of Gene Sequencing Analysis, Department of Applied Genomics, Kazusa DNA Research Institute, Chiba, Japan

**Keywords:** Macrophages, Liver fibrosis, Interleukin 34, Interleukin 4, Alternative activated macrophage

## Abstract

**Background:**

For the treatment of liver fibrosis, several novel cell therapies have been proposed. Autologous macrophage therapy has been reported as one of the promising treatments. So far, most studies have used colony-stimulating factor 1 (CSF-1) to induce the differentiation of macrophage progenitor cells. The receptor for CSF-1, CSF-1R possesses another ligand, interleukin 34. However, the therapeutic capacity for liver fibrosis by interleukin 34-induced macrophages has not been evaluated.

**Methods:**

We have employed acute (bile duct ligation) and chronic (administration of carbon tetrachloride or thioacetamide) liver fibrosis models. Using these models, we evaluated the therapeutic capacity of macrophages induced by interleukin 34-based conditions. In most experiments, interleukin 4 was also added to the differentiation process to induce alternative-activated macrophages. As a mechanism analysis, we have examined liver inflammation and damage, the status of stellate cells, and the immunosuppressive capacity of the macrophages. Human macrophages were differentiated from CD14^+^ monocytes and analyzed.

**Results:**

In both acute and chronic liver damage experiments, interleukin 34-induced macrophages significantly ameliorated liver fibrosis. The addition of interleukin 4 to the differentiation process resulted in an increase of obtained macrophages and a bias to alternative activated macrophages (so-called M2). The alternative activated macrophages (M2-type) showed a reproducible therapeutic effect of liver fibrosis with a suppression of parameters of liver inflammation and damage, stellate cells, and T cell activation. Similar macrophages could be differentiated from human CD14^+^ monocytes in the presence of interleukin 34 plus interleukin 4, and a therapeutic effect was observed using a humanized mouse model.

**Conclusions:**

Interleukin 34-induced macrophages, particularly when additionally stimulated with interleukin 4, significantly ameliorated the liver fibrosis.

**Supplementary Information:**

The online version contains supplementary material available at 10.1186/s41232-025-00364-7.

## Background

Liver fibrosis is induced by various causes including hepatitis B, hepatitis C, fatty liver, and alcoholic hepatitis. Persistent inflammation over extended periods results in fibrosis in the liver, evolving into a state known as cirrhosis [1]. Reaching a decompensated stage of cirrhosis often leads to fatal conditions such as hepatocellular carcinoma. Currently, the only curative treatment for cirrhosis is liver transplantation. Liver transplantation ranks as the second most frequent solid organ transplant, but the present rate of transplantation addresses less than 10% of the worldwide demand for transplants [2]. Consequently, in fibrotic liver diseases, it is essential to provide treatments and management that prevent the condition from deteriorating to the point where liver transplantation becomes necessary. However, as of now, there is no treatment that “actively” suppresses or improves liver fibrosis. For cirrhosis, novel treatments involving the use of cells from an individual or a donor have been developed. This includes a variety of cell types like endothelial progenitor cells or mesenchymal stem cells [3]. Among these options, a straightforward method has been suggested, termed autologous macrophage (Mf) therapy. In this approach, Mfs are cultured and differentiated from a patient’s peripheral blood cells and then reintroduced into the patient. A first-in-human, phase 1 dose-escalation trial assessing the safety of this technique has been carried out, with results indicating not only its safety but also encouraging therapeutic outcomes as secondary endpoints [4].

Mfs differentiate and survive in response to signals from colony stimulation factor-1 receptor (CSF-1R). CSF-1 and interleukin 34 (IL-34) have been identified as ligands that bind to CSF-1R [5]. While the molecular homology between CSF-1 and IL-34 is limited, both IL-34 and CSF-1 exhibit similar functions in modulating differentiation, proliferation, and survival of the myeloid lineage [6, 7]. While CSF-1 is ubiquitously expressed throughout the body, IL-34 has a limited expression in physiological conditions. IL-34 is primarily produced by keratinocytes and neurons and plays a pivotal role in the differentiation and survival of Langerhans cells and microglia [8]. On the other hand, we have identified that IL-34 is highly expressed in various malignant tumors [7, 9–11]. We found that IL-34 contributes to tumor growth by generating immunosuppressive Mfs in the tumor microenvironment, which also plays a role in resistance to chemotherapy, radiotherapy, and immunotherapy [12–14]. Leveraging these insights, we turned our attention to IL-34 as an inducing cytokine for Mf therapy targeting liver fibrosis diseases.

Mfs are roughly classified into M1- and M2-Mfs; to simplify and summarize, M1-Mfs induce inflammation, thereby promoting tissue injury, whereas M2-Mfs, which are activated as part of the type 2 immunity regulated by IL-4 or IL-13, have anti-inflammatory effects and are involved in tissue repair and regeneration [15–17]. Therefore, the latter is considered to be overlapped with alternatively activated or wound-healing Mfs [18, 19]. For successful liver fibrosis treatment, strategies to deliver Mfs that have anti-inflammatory and tissue-repairing functions are considered to be important.

In this study, we investigated the capacity of IL-34 Mf to improve liver fibrosis. Furthermore, we also investigated the effect of alternatively activated Mf induced by IL-34 + IL-4. The results indicate that Mf induced by IL-34-based conditions has a substantial capacity to ameliorate liver fibrosis. This study suggests an alternative IL-34-using protocol to generate autologous Mf for the treatment of liver fibrosis.

## Methods

### Mice

Male 6-week-old C57BL/6 mice were purchased from Japan SLC, Inc. (Shizuoka, Japan). Male 5 weeks aged NOD. Cg-*Prkdc*^*scid*^* Il2rg*^*tm1Wjl*^/SzJ (NSG mice) were purchased from Jackson Laboratory Japan. All animal procedures were approved by the Hokkaido University Animal Care Committee (approval number: 20–0086). All mice were housed at 25 °C under a 12-h light–dark cycle, with darkness from 9:00 PM to 9:00 AM. Water and standard chow were provided ad libitum.

### Mf culture

Mouse Mf culture medium was RPMI-1640 (Fujifilm Wako Pure Chemical Industries) supplemented with 10% fetal bovine serum (Sigma), 1% penicillin/streptomycin (Nacalai Tesque), and 1% non-essential amino acid (Nacalai Tesque). To induce mouse Mfs, 5 × 10^6^ mouse bone marrow cells were suspended in 10 ml of Mf medium and cultured in a 10-cm dish for 6 days in the presence of 50 ng/ml of IL-34 or CSF-1 (BioLegend). In some experiments, 10 ng/ml of IL-4 (BioLegend) was added from day 0. All cells were maintained in a humidified incubator with 5% CO_2_ at 37 °C. The viable cell count of Mf was analyzed using an MTT Cell count kit (Nacalai Tesque). Absorbance at a test wavelength of 570 nm and a reference wavelength of 650 nm was measured by using a Multiskan FC (Thermo Fisher Scientific). To induce human Mfs, CD14^+^ monocytes were magnetically sorted using CD14 MicroBeads (Miltenyi Biotec) from peripheral blood mononuclear cells (PBMCs). 1 × 10^6^ sorted cells were suspended in 2 ml of TexMACS™ Medium (Miltenyi Biotec) and cultured in a 3.5-cm dish for 6 days in the presence of 100 ng/ml of IL-34 (BioLegend) and 10 ng/ml of IL-4 (Miltenyi Biotec) from the beginning of the culture. For human study, the procedures were approved by the Hokkaido University Hospital Committee (approval number: 22–0020).

### Induction of liver fibrosis and treatment by Mf

For the induction of chronic liver fibrosis, two experimental models were employed. First, mice were given sterile water containing 0.03% thioacetamide (TAA) including 0.1% sucralose by free drinking. Notably, during the initial week, the concentration was adjusted to 0.015% thioacetamide in the drinking water. Second, 20 μl of carbon tetrachloride (CCl_4_) was dissolved in 180 μl of corn oil and administered intraperitoneally to mice twice weekly.

For the induction of acute liver damage and fibrosis, bile duct ligation (BDL) was performed following the methodology outlined by Tag et al. [20]. Briefly, mice were anesthetized for pain relief and sedation, and a midline abdominal incision was made. The liver was carefully flipped to expose the bile duct, which was then isolated from the adjacent blood vessels. The bile duct was ligated in three places using surgical sutures. The liver was then repositioned, and the peritoneum and skin were sutured and closed. Mice were kept warm until they regained consciousness.

For the experiments for human Mf, NSG mice were administered with 1 × 10^7^ human PBMCs to reconstitute the human immune condition. In our preliminary experiments, we further gave TAA to the NSG mice administered with human PBMCs. However, in this condition, all the mice died showing severe wasting syndrome. Therefore, we decided not to give the hepatotoxic agents further. On the other hand, we found that, in the NSG mice administered with human PBMCs alone, graft versus host disease-like xenogeneic reaction was induced, and 8 weeks later, liver fibrosis could be detected (Fig. 5C). Then, in this study, we used this model for examining the therapeutic effect of human Mf.

For therapeutic Mf administration, 2 × 10^6^ Mfs were suspended in saline and carefully administered via the tail vein at the timing indicated in figure legends. For the humanized model (using NSG mice), 2 × 10^6^ Mfs were intravenously administered at 4 weeks after the PBMCs injection, and at 8 weeks liver and blood samples were obtained and examined.

### Measurement of aspartate aminotransferase (AST) and alanine aminotransferase (ALT)

Sera were collected from whole blood by centrifugation at 3000 rpm for 10 min at 4 ℃. AST and ALT were analyzed by SRL Laboratory (Sapporo). The timing of blood sample collection is indicated in figure legends.

### Sirius red and terminal deoxynucleotidyl transferase-mediated dUTP nick end labeling (TUNEL) staining

Sirius red solution was purchased from Muto Pure Chemicals Co., Ltd. (Tokyo, Japan). Sirius red staining was performed according to the method described by Junqueira et al. [21]. Specimen images were captured using a microscope (Keyence Corporation) after Sirius red staining. For more than 10 fields of view per liver sample, the fibrotic areas were quantified using Image J software (NIH). The proportion of fibrotic areas within the captured field was calculated. To detect apoptotic cells, a TUNEL assay was performed using in situ Apoptosis Detection Kit™ (Takara). For more than 10 fields of view per liver sample, TUNEL-positive cells were quantified using ImageJ software (NIH).

### Flow cytometry

Flow cytometry was performed using BD FACSCelesta™ (BD Biosciences, Franklin Lakes, NJ, USA) and data were analyzed using FlowJo software (Tree Star, Ashland, OR, USA) or Kaluza (Beckman Coulter). Fluorescence-labeled monoclonal antibodies and corresponding isotype controls were purchased from BioLegend otherwise indicated. The antibodies were anti-mouse F4/80 (clone; BM8), CD206 (C068C2), I-A/I-E (M5/114.15.2), H-2 D^b^/K^b^ (KH95), PD-L1 (10F.9G2), PD-L2 (TY25), CD3 (145-2C11), CD4 (RM4-5), and CD8 (53–67), and anti-human CD206 (19.2, BD Bioscience), HLA-DR (L243), HLA-ABC (W6/32, Invitrogen), PD-L1 (29E.2A3), PD-L2 (24F.10C12), CD4 (OKT4, eBioscience), and CD8 (SK1). For analysis, live cells were gated based on forward and side scatter as well as lack of DAPI, propidium iodide, or 7-AAD uptake. All antibodies were used at 1∶200 dilutions. For T cell and Mf coculture experiments, T cells were magnetically sorted from mouse spleen or human PBMCs using CD90.2 Microbeads, mouse or Pan T Cell Isolation Kit, and human (Miltenyi Biotec), respectively. The T cells were labeled with carboxyfluorescein succinimidyl ester (CFSE) and stimulated with anti-CD3 and CD28 antibodies (BioLegend) for mice or with Dynabeads Human T-Activator CD3/CD28 (Gibco) for human for 4 days. Mfs were co-cultured with the CFSE-stained T cells from the beginning of the culture. After the coculture, the fluorescence intensity of CFSE and cell count for cells gated on CD4 and CD8 were examined by flow cytometry.

### Immunohistochemical analysis

The liver samples were fixed in 4% paraformaldehyde (Fujifilm Wako Pure Chemical) for 24 h at 4 °C and then embedded in paraffin. The embedded samples were sliced, and 5 µm thick sections were obtained. Sections were deparaffinized, and endogenous peroxidase was blocked by 0.3% H_2_O_2_ in distilled water for 20 min. Sections were then incubated with BlockAce (DS Pharma Biomedical) in PBS for 1 h to block non-specific reactions. After protein blocking, the sections were incubated with anti-COL1A1 (E8F4L, CST), α-SMA (1A4, BioLegend), CD8 (4SM15, Invitrogen), or CD69 (H1.2F3, BioLegend) antibody in PBS overnight at room temperature. After washing, the sections were incubated with horse radish peroxidase-linked secondary antibody (BioLegend) for 1 h at room temperature, followed by incubation with 3, 3′-diaminobenzidine tetrahydrochloride (Fujifilm Wako Pure Chemical) in Tris–HCl containing H_2_O_2_ for 5–20 min and counterstained with hematoxylin. Sections were then enclosed using Marinol (Muto Pure Chemicals Co., Ltd.). The observation was performed using a light microscope BX53F (Olympus). For more than 10 fields of view per liver sample, the immune-stained areas or cells were quantified using ImageJ software (NIH).

### RNA sequencing analysis

RNA was extracted using the NucleoSpin RNA kit (TakaraBio). Subsequently, cDNA was synthesized from the RNA using the SureSelect Strand-Specific RNA Library Prep Kit (Agilent Technologies). For the preparation of samples for sequence analysis, the NextSeq 500/550 High Output Kit v2.5 (75 cycles) (Illumina) was utilized. Sequencing was performed using the NextSeq 500 platform (Illumina). Fastq files were generated from the obtained reads using the bcl2fastq program (Illumina).

### Statistics

JMP software (JMP Version 16.0.0, SAS Institute Inc.) and R (version 4.2.3.) were used for statistical analysis. Data represent mean ± SEM. The Student's *t*-test (unpaired, two-tailed) or Tukey’s honest significant difference (HSD) test was used to test for statistically significant differences.

## Results

### Mfs induced by IL-34 from bone marrow cells inhibit liver fibrosis

**Fig. 1:**
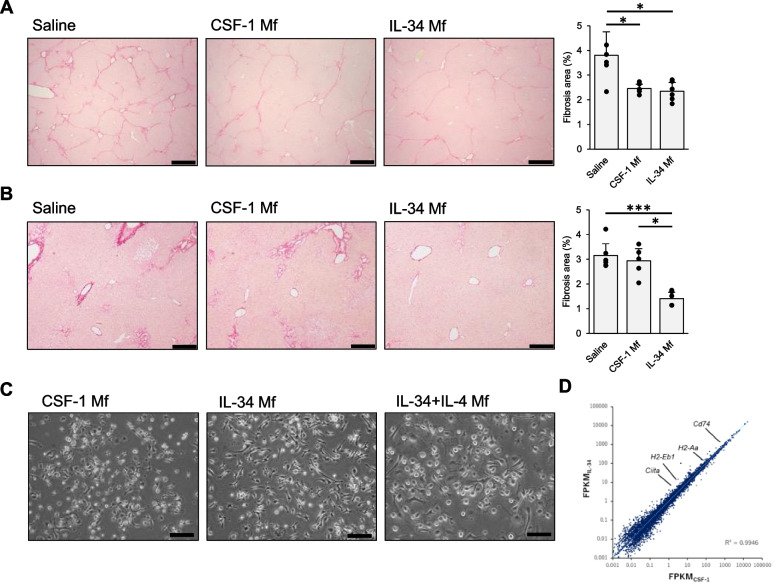
IL-34-induces Mf from bone marrow cells and inhibits liver fibrosis. **A**, **B** Sirius red staining of liver sections from C57BL/6 J mice with liver fibrosis induced by CCl_4_ (**A**) and BDL (**B**). Similar results were obtained from two independent experiments. **A** Saline or Mfs were intravenously injected at 8 weeks, and at 12 weeks liver samples were obtained (*n* = 6 each group). **B** Saline or Mfs were intravenously injected at 10 days, and at 14 days liver samples were obtained (*n* = 6 each group). Scale bar indicates 200 μm. The bar graphs represent the quantified Sirius red-positive area ratio (mean ± SEM). *P < 0.05; ***P < 0.001. **C** Appearance of the Mfs. Scale bar indicates 100 μm. **D** Comparison of mRNA expressions of CSF-1 and IL-34 Mfs

It has been reported that CSF-1-induced murine bone marrow cell-derived Mfs intravenously administered from tail vein ameliorate carbon tetrachloride (CCl_4_)-induced liver fibrosis [22]. Similarly, we induced Mfs from mouse bone marrow cells using IL-34 and administered them to mice with CCl_4_-induced liver fibrosis. The results showed that fiber deposition was inhibited to the same extent as CSF-1 Mfs at 4 weeks after Mf administration (Fig. 1A). Next, we performed an acute liver damage model with bile duct ligation (BDL). In this experiment, CSF-1 Mfs showed minimal effect, whereas IL-34 Mfs inhibited fibrosis (Fig. 1B). Thus, the administration of IL-34 Mf has potential in the treatment of liver fibrosis.

**Fig. 2:**
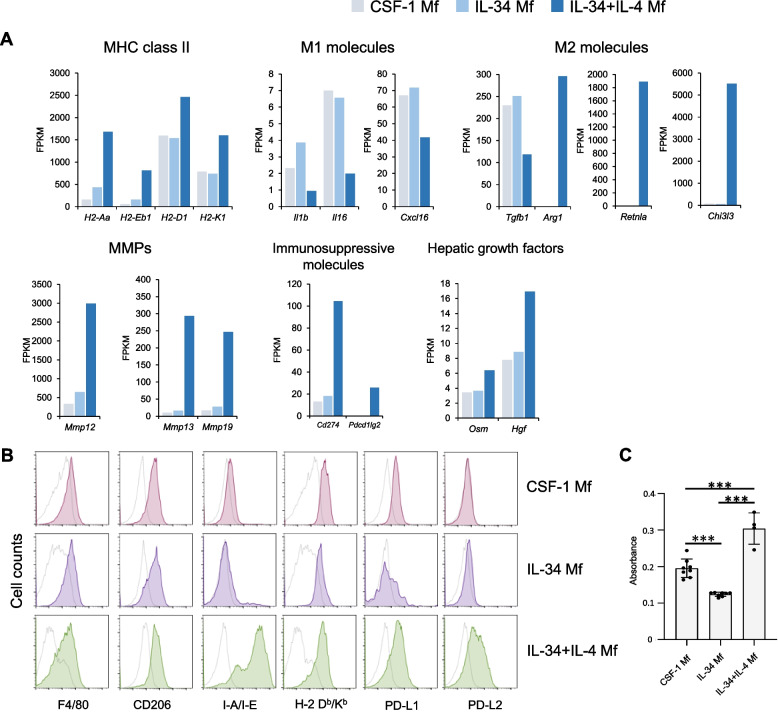
IL-34 + IL-4-induced Mf exhibits M2-biased, immunosuppressive properties. **A** Comparison of mRNA expression levels of Mfs induced by each cytokine, shown as fragments per kilobase of exon per million mapped reads (FPKM). **B** Flow cytometry analyses. Colored histograms showing cells expressing the indicated surface proteins. White histograms showing the isotype control staining. Similar results were obtained from more than two independent experiments. **C** Cell viability of Mfs detected by MTT assay. ***P < 0.001

We compared the appearance and gene expression of CSF-1 and IL-34 Mfs. The appearance of the Mfs is similar, both round, partly spindle-shaped, and partly attached to a plastic plate (Fig. 1C). Next, mRNA expression was analyzed by RNA sequencing. As shown in Fig. 1D, approximate expression patterns are consistent. Looking at the detail, IL-34 Mf showed enhanced expression of MHC class II-related genes such as *H2-Aa*, *H2-Eb1*, *Cd74*, and *Ciita* compared with CSF-1-induced Mf. The expression of these genes may be related to enhanced MHC class II protein expression when IL-4 was added to the IL-34 Mf differentiation culture, which is shown later (Fig. 2B).

Therefore, IL-34 Mf is similar to CSF-1 Mf. The therapeutic capacity of IL-34 Mf for liver fibrosis seems consistent to, or at least not inferior to, that of CSF-1 Mf.

### IL-34 + IL-4 induced Mfs exhibit M2-biased, immunosuppressive properties

Next, we alternatively activated IL-34 Mfs by the addition of IL-4 to the differentiation culture from bone marrow cells. The appearance of IL-34 + IL-4-induced Mfs seemed more spindle-shaped than those induced with IL-34 alone and strongly adhered to the plastic surface (Fig. 1C).

RNA sequencing was performed to examine the mRNA expressions of induced Mfs (Fig. 2A). In the IL-34 + IL-4-induced Mfs, the expression of MHC-related genes was upregulated. The expression of *Il1b*, *Il16*, and *Cxcl16*, markers of M1-type Mf, was reduced by about 40%, while *Arg1*, *Retnla*, and *Chi3l3*, markers of M2-type Mf, were expressed at exceptionally high levels. These findings suggest that IL-34 + Il-4-induced Mf polarized towards the M2 type. On the other hand, the expression level of *Tgfb1*, a gene specific to type M2 and promoting fibrosis by activating hepatic stellate cells, was rather downregulated. Some matrix metalloproteinases (MMP), such as *MMP 12*, *13*, and *19* were highly upregulated. *Cd274* (PD-L1) and *Pdcd1Ig2* (PD-L2), both related to immunosuppression were also upregulated in IL-34 + IL-4-induced Mf. Several hepatic growth factors such as *Osm* (Oncostatin M) or *Hgf* (hepatocyte growth factor) were also upregulated in IL-34 + Il-4 Mf. Gene expressions in Mfs induced with CSF-1 + IL-4 were also examined with quantitative RT-PCT. The CSF-1 + IL-4 Mfs showed similar gene expressions with those of IL-34 + IL-4 Mfs (Fig. S1A).

Several cell surface markers were analyzed with flow cytometry (Fig. 2B). The results showed that Mf markers such as F4/80 or CD206 (one of the M2 markers) were expressed in every Mf. Expression of MHC class II was enhanced in IL-34 + IL-4-induced Mf than others as described above. PD-L1 and PD-L2 are known to bind to PD-1 and negatively regulate T cells by acting in a suppressive manner against MHC-mediated TCR signaling [23]. In the IL-34 + IL-4 Mf, although PD-L1 expression was not altered, PD-L2 expression was apparently upregulated. These results suggest that IL-34 + IL-4 Mf may negatively regulate T cells through PD-1 suppressive signals. Mfs induced with CSF-1 + IL-4 were also examined. The CSF-1 + IL-4 Mfs showed a similar FACS pattern to that of IL-34 + IL-4 Mfs (Fig. S1B).

In addition, cell proliferative capacity was examined using MTT Assay. The results showed that IL-34 + IL-4 Mf had significantly higher cell proliferative capacity than the CSF-1 and IL-34 groups (Fig. 2C). The ability to obtain a higher number of cells using similar culture protocols is considered one of the advantages in developing autologous Mf therapies.

### IL-34 + IL-4 induced Mfs prevent both acute and chronic liver fibrosis models

**Fig. 3:**
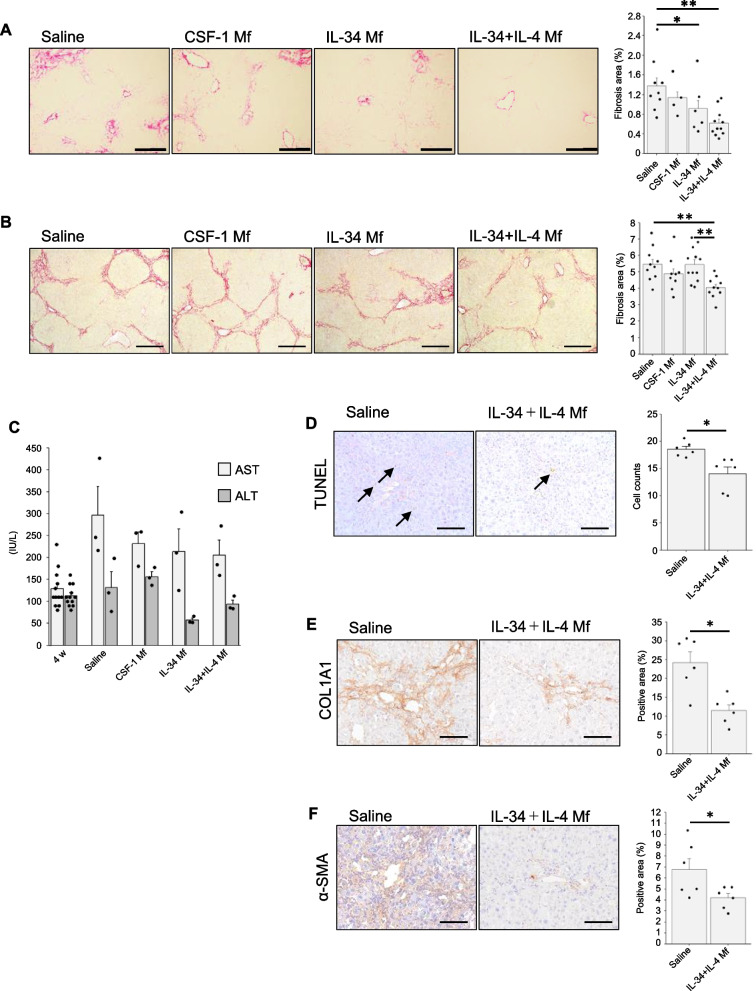
IL-34 + IL-4-induced Mf prevents both acute and chronic liver fibrosis models. **A** Sirius red staining of liver sections from BDL-induced liver fibrosis mice (*n* > 6 each group). The timing of treatment and sample collection was the same as Fig. 1B. Scale bar indicates 200 μm. **B** Sirius red staining of liver sections from TAA-induced liver fibrosis mice (*n* > 6 each group). Saline or Mfs were intravenously injected at 6 weeks, and at 10 weeks liver samples were obtained. Scale bar indicates 200 μm. Bar graphs show the quantification of Sirius red-positive area ratios (mean ± SEM). Similar results were obtained from two independent experiments. **C** AST and ALT levels in the sera of the TAA model (mean ± SEM). Blood samples were collected before (at 4 weeks) and after (at 10 weeks) the Mf treatment. **D** TUNEL staining of the liver samples at 10 weeks (TAA model). Bar graphs show the TUNEL-positive cells. Bar graphs show the cell counts of TUNEL-positive cells (mean ± SEM). **E**, **F** Immunohistochemistry of liver sections for COL1A (**C**) and α-SMA (**D**) expressions in the TAA model. Bar graphs show the quantification of immunostained positive area ratios (mean ± SEM). * *P* < 0.05; **P < 0.01

Next, we investigated whether treatment with the alternatively activated Mf induced by IL-34 + IL-4 was also effective in liver fibrosis models. At first, we confirmed the therapeutic effect of them in the acute liver damage model with BDL. In this experiment, IL-34 + IL-4-induced Mf showed the strongest potential to treat liver fibrosis among the Mfs examined (Fig. 3A). In the chronic liver damage model with TAA, the therapeutic effect of Mfs induced with CSF-1 or IL-34 alone was limited. In contrast, the IL-34 + IL-4 Mfs significantly suppressed liver fibrosis compared to the saline or IL-34 alone group (Fig. 3B). AST and ALT levels in the sera of the TAA model, representing hepatocyte damage, showed a tendency to decrease with the Mf treatments, although there was no statistically significant difference. (Fig. 3C).

We then focused on the effect of IL-34 + IL-4 Mf in the formation of hepatic fibrosis in the TAA model. It has been suggested that liver parenchymal cell damage induces inflammation in the liver which in turn activates hepatic stellate cells producing fibrous tissues [17]. TUNEL staining of the liver samples indicating apoptotic cell death showed that the IL-34 + IL-4 Mf treatment significantly decreased the number of TUNEL-positive cells (Fig. 3D). This result suggests that the IL-34 + IL-4 Mf treatment contributes to the protection from liver damage. Next, we examined the effect of IL-34 + IL-4 Mf on the hepatic stellate cells themselves. The expression of type I collagen alpha 1 (COL1A1), the main component of liver fibers produced by activated hepatic stellate cells, was evaluated with immunohistochemistry. As expected, the expression of COL1A1 was significantly reduced with the IL-34 + IL-4 Mf treatment (Fig. 3E). The expression of α-SMA, the marker of stellate cell activation, was also examined, which was significantly reduced with IL-34 + IL-4 Mf treatment (Fig. 3F). Therefore, it is suggested that, as a mechanism, IL-34 + IL-4 Mf treatment protected the liver from hepatotoxic damage, suppressed hepatic stellate cell activation, and reduced the fibrotic change.

### IL-34 + IL-4 Mf suppresses T cell responses

**Fig. 4:**
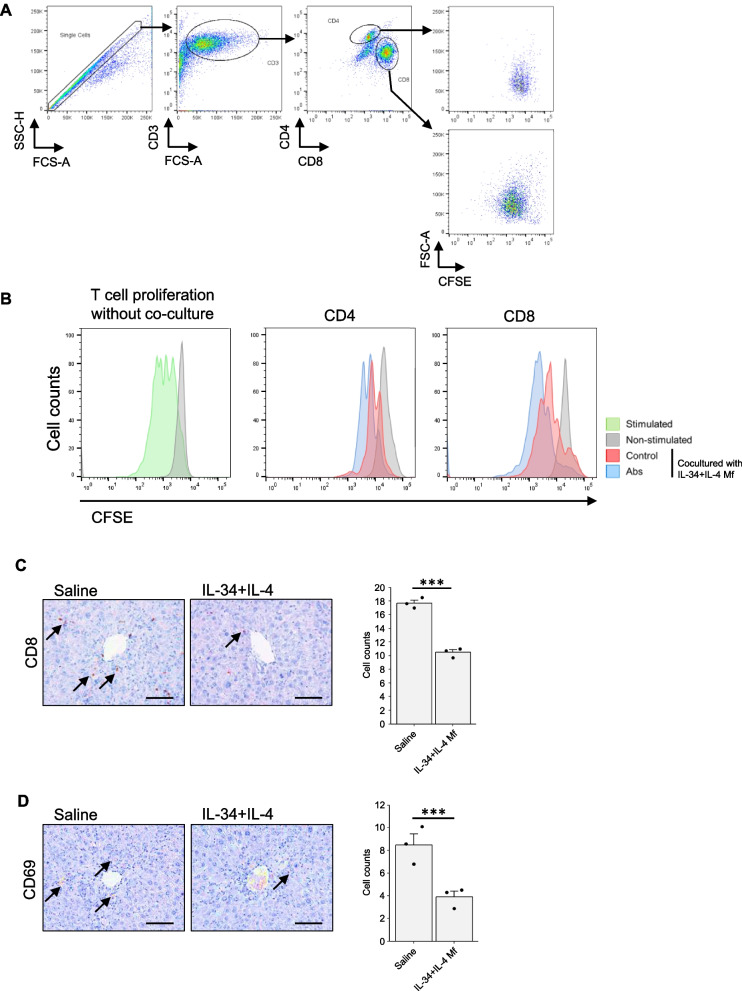
IL-34 + IL-4-induced Mf suppresses T cell reponses. **A** Gating strategy of the flow cytometry analysis. **B** IL-34 + IL-4 Mfs were co-cultured with CFSE-stained T cells, separated from spleen cells by magnetic cell sorting, and stimulated with anti-CD3 and CD28 antibodies for 4 days. The histogram shows CFSE fluorescence intensity for cells gated on CD4- and CD8-positive cells. Culture conditions include stimulated T cells without co-culture (stimulated; green), T cells without proliferation stimulation (non-stimulated; gray), and co-cultured with Mf in the presence of control IgG (control; red) or anti-PD-L1 and anti-PD-L2 antibodies (Abs; blue). Similar results were obtained from two independent experiments. **C**, **D** Immunohistochemistry of CD8- or CD69-positive cells in the liver sections from TAA-induced liver fibrosis mice. Bar graphs show the quantification of immunostained cell counts (mean ± SEM, *n* = 3 each group). ***P < 0.001

As T cells, particularly CD8-positive ones, are known to activate hepatic stellate cells and promote fibrotic protein production by secreting inflammatory cytokines [24], we next examined the ability of IL-34 + IL-4 Mf to suppress T cell activation. Spleen-derived T cells were first labeled with CFSE and stimulated with anti-CD3 and CD28 antibodies. Then the T cells were co-cultured with IL-34 + IL-4 Mfs in the presence or absence of blocking antibodies against PD-L1 and PD-L2. Four days later, T cell proliferation was analyzed with flow cytometry. The FACS gating strategy is shown in Fig. 4A. As shown in Fig. 4B. the histogram indicating either CD4 or CD8 T cell division was attenuated by co-culturing with IL-34 + IL-4 Mf (Control). On the other hand, when blocking antibodies against PD-L1 and PD-L2 were added to the culture, the weakened CFSE peak shifted to the mitogenic side (Abs). These results indicate that IL-34 + IL-4 Mf inhibits the proliferation of CD4 and CD8 T cells via PD-1-mediated inhibitory signal. When similar experiments were performed using CSF-1 + IL-4 Mf, a comparable inhibitory effect was observed upon CD4 and CD8 T cell proliferation (Fig. S1C). We then examined in vivo CD8 T cells in the liver of the TAA-induced fibrosis model treated with IL-34 + IL-4 Mf. In line with the in vitro results, cell counts of CD8 T cells in the liver were significantly reduced in the IL-34 + IL-4 Mf-treated group (Fig. 4C). Lymphocyte activation was also assessed by staining of CD69. The results indicated that the IL-34 + IL-4 Mf treatment significantly suppressed the lymphocyte activation in the liver (Fig. 4D). Although it is currently unclear whether these effects are due to inhibition of CD8 T cell migration into the liver, or inhibition of their proliferation in the liver, IL-34 + IL-4 Mf seemed to exhibit the therapeutic effect through suppression of CD8 T cells (and also other lymphocytes) as one of the mechanisms.

### Human IL-34 + IL-4 induced Mf shows similar phenotype and function with mouse one

**Fig. 5:**
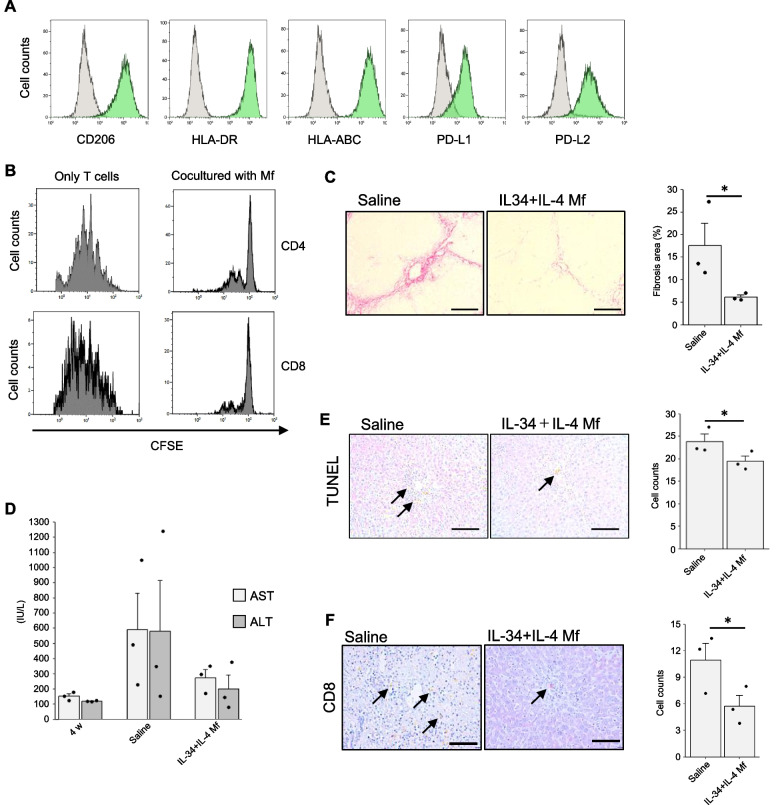
Phenotype and function of human IL-34 + IL-4-induced Mf. **A** Flow cytometry analyses. Green histograms showing cells expressing the indicated surface proteins. Gray histograms showing the isotype control staining. **B** Human IL-34 + IL-4 Mfs were co-cultured with CFSE-stained T cells stimulated with anti-CD3 and CD28 antibodies for 4 days. The histogram shows CFSE fluorescence intensity for cells gated on CD4 and CD8. **C** Sirius red staining of liver sections from human PBMCs-injected NSG mice (*n* = 3 each group). Saline or IL-34 + IL-4 Mfs were intravenously injected at 4 weeks, and at 8 weeks liver samples were obtained. Scale bar indicates 200 μm. Bar graphs show the quantification of Sirius red-positive area ratios (mean ± SEM). **D** AST and ALT levels in the sera (mean ± SEM). Blood samples were collected before (at 4 weeks) and after (at 8 weeks) the Mf treatment. **E**, **F** TUNEL or CD8 staining of the liver samples. Bar graphs show the counts of positive cells (mean ± SEM). *P < 0.05

Finally, we attempted to differentiate human Mfs. Human peripheral blood CD14^+^ cells were cultured in the presence of IL-34 + IL-4. Flow cytometry analysis showed that human IL-34 + IL-4 Mfs highly expressed CD206, one of M2 markers (Fig. 5A). They also highly expressed HLA class I and II, PD-L1 and PD-L2 (Fig. 5A), as mouse Mfs did (Fig. 2B). Mfs induced with human CSF-1 + IL-4 Mf showed similar FACS pattern with that of human IL-34 + IL-4 Mfs (Fig. S1D). When cocultured with human T cells stimulated with anti-CD3 and CD28 antibodies, human IL-34 + IL-4 Mf significantly inhibited the CFSE peak shift to the mitogenic side, thus they effectively suppressed the T cell activation (Fig. 5B). Finally, we examined the therapeutic effect of human IL-34 + IL-4 Mf in a liver fibrosis model using immunodeficient NSG mice in which human PBMCs had been administered (see the *Methods* section). Eight weeks after the PBMCs injection, apparent fibrosis was observed in the control group (Fig. 5C). In the treatment group, IL-34 + IL-4 Mfs were administered at 4 weeks, and a further 4 weeks later liver and blood samples were examined. As shown in Fig. 5C, liver fibrosis was significantly inhibited with the IL-34 + IL-4 Mf treatment. AST and ALT levels in the sera showed a tendency to decrease with the IL-34 + IL-4 Mf treatment, although there was no statistically significant difference (Fig. 5D). Intrahepatic counts of apoptotic cells and CD8 T cells were significantly reduced in the IL-34 + IL-4 Mf-treated group (Fig. 5E, F). It is therefore likely that human IL-34 + IL-4-induced Mfs are as effective against liver fibrosis as in mice.

## Discussion

In this study, Mfs induced with IL-34-based conditions were shown to be effective in the treatment of liver fibrosis in mouse models. In particular, the addition of IL-4 to IL-34 in the Mf differentiation process polarizes the cells into an anti-inflammatory M2-type phenotype and improves the obtained cell numbers, making it a promising material for cell therapy. In various studies to date, different types of cellular therapies have been shown to be effective in the treatment of liver fibrosis. Recently, transplantation of iPS cell-derived liver organoids was shown to reduce chemically induced liver fibrosis [25]. However, in this case, changes in the microenvironment due to increased M2-type Mfs have been shown to be important. There are also other reports that the final effector cells in cell therapy for liver fibrosis are M2 or wound healing Mfs [15, 26]. Therefore, it would be a simpler and easier way to generate and administer autologous M2 Mf without the need to create complicated cells. The primary aim of this study was to investigate the effects of Mf induced by IL-34-based conditions on liver fibrosis. Therefore, we did not actively examine Mf induced by CSF-1, particularly CSF-1 + IL-4 Mf. It is already well-established that CSF-1 Mf is highly effective against liver fibrosis [4, 22]. While it is conceivable that Mf induced by CSF-1 with the addition of IL-4 would also exhibit sufficient functionality, further studies are required to verify this.

The mechanism of the Mf therapeutic effect on liver fibrosis has not been fully elucidated. However, CD8-positive T cells are known to produce TNF-α and IFN-γ in the liver in an inflammatory environment and to enhance TGF-β production in the surrounding tissues [27, 28]. TGF-β activates hepatic stellate cells to secrete extracellular matrix protein COL1A1, which in turn induces liver fibrosis. In the present study, IL-34 + IL-4 Mf treatment reduced the activity of hepatic stellate cells (Fig. 3E, F), without upregulating TGF-β expression (Fig. 2A), suggesting that one of the protective mechanisms of IL-34 + IL-4 Mf is the suppression of hepatic stellate cells. Besides suppressing hepatic stellate cells, IL-34 + IL-4 Mfs significantly suppressed liver parenchymal cell damage (Figs. 3D and 5E) and T cell activation (Figs. 4B and 5B), which is upstream of the liver fibrosis process. Furthermore, a previous report suggests that administered Mfs dissolves collagen fibers by secreting MMPs [22]. In this study, IL-34 + IL-4 Mfs were shown to highly express MMP12 and 13 (Fig. 2A), which may have a similar anti-fibrotic effect. Therefore, mechanisms of liver fibrosis inhibition by IL-34 + IL-4 Mf may function in a complex manner at multiple points.

PD-L2 is known to be regulated by the transcription factor Stat6. It has been reported that co-incubation of CSF1 + IL-4-induced Mf from Stat6 KO mice with activated T cells resulted in de-repression of CD4 and CD8 positive T cell growth, suggesting an inhibitory role of PD-L2 on Mfs [29]. Furthermore, it is reported that MHC class II molecules are also transcriptionally regulated by Stat6, and the expression of MHC class II molecules is not increased in CSF1 + IL-4-induced Mf from Stat6 KO mice [29]. In the present study, it was shown that inhibition of PD-L2 on Mfs by an antibody can recover T-cell suppression, a result consistent with the above report (Fig. 4B). Furthermore, the expression of MHC class II was apparently increased in IL-34 + IL-4 Mf (Figs. 2B and 5A). Therefore, it is possible that Stat6 similarly regulates the phenotype and function of IL-34 + IL-4 Mf in the treatment of liver fibrosis at the transcription factor level. It would be interesting to investigate this point in the future study. Although the functional role of upregulated MHC class II on Mfs has not been clarified in this study, the high expression would be used for a marker of Mfs induced with IL-34 + IL-4.

Limitations of the present study include insufficient analysis of regulatory T cells (Tregs). It has been reported that, whereas Tregs activate hepatic stellate cells via secretion of IL-8 to enhance liver repair in the early phase of liver inflammation, they exert an immunosuppressive and tissue-protective function in prolonged liver inflammation [30]. Tregs suppress CD4, CD8-positive effector T cells and may function synergistically with administered Mfs. In liver fibrosis associated with prolonged inflammation, clarifying the association of Mf treatment with Treg function could elucidate the therapeutic mechanism in the course of hepatitis.

## Conclusions

In this study, we clearly indicated that IL-34-induced Mf, particularly when alternatively activated by the addition of IL-4, significantly ameliorated liver fibrosis in mouse models. As a mechanism, suppression of liver inflammation and damage as well as stellate cell and T cell activation was suggested. These results support the development of novel cell therapy for liver fibrosis with autologous Mfs induced by IL-34-based conditions.

## Supplementary Information

Supplementary Material 1: Figure S1. Phenotype and function of CSF-1 + IL-4 Mf.

## Data Availability

The datasets used and/or analyzed during the current study are available from the corresponding author on reasonable request.

## Abbreviations

Mf: Macrophage
CSF: Colony stimulation factor
IL: Interleukin
NSG: NOD.Cg-*Prkdc*^*scid*^* Il2rg*^*tm1Wjl*^/SzJ
TAA: Thioacetamide
CCl_4_: Carbon tetrachloride
BDL: Bile duct ligation
PBMCs: Peripheral blood mononuclear cells
CFSE: Carboxyfluorescein succinimidyl ester
MMP: Matrix metalloproteinases
COL1A1: Type I collagen alpha 1
SMA: Smooth muscle actin
