# Supplementary material for: Amelioration of liver fibrosis with autologous macrophages induced by IL-34-based condition
Source: Inflamm Regen. 2025 Jan 24;45:2. doi: 10.1186/s41232-025-00364-7 (PMC11758727; doi:10.1186/s41232-025-00364-7)
Supplement: Supplementary file 1 — Supplementary Material 1: Figure S1. Phenotype and function of CSF-1 + IL-4 Mf. [file 41232_2025_364_MOESM1_ESM.pptx]

## Slide 1
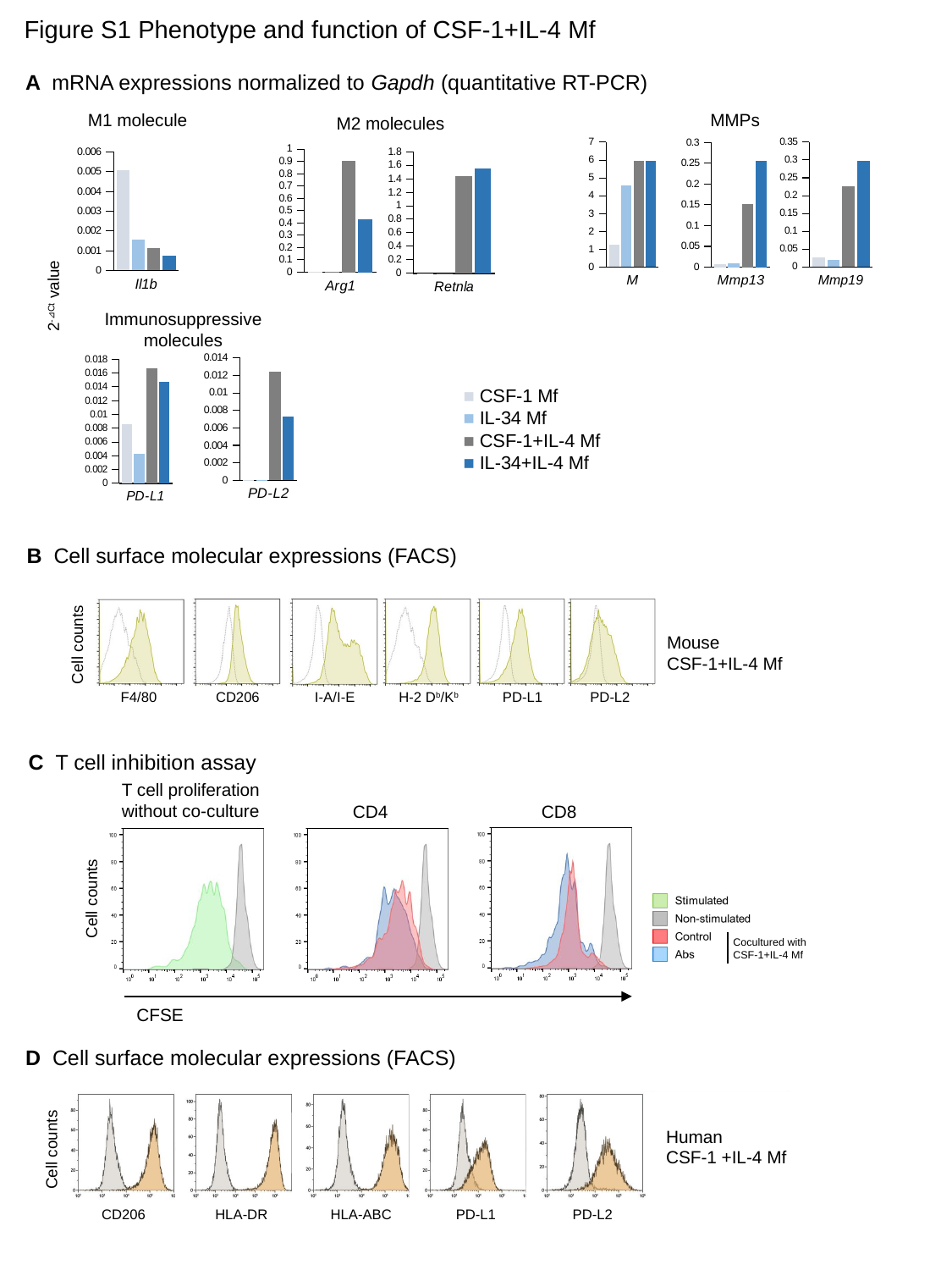

Figure S1 Phenotype and function of CSF-1+IL-4 Mf
A mRNA expressions normalized to Gapdh (quantitative RT-PCR)
MMPs
M1 molecule
M2 molecules
### Chart
| Category | CSF-1 Mφ | IL-34 Mφ | CSF-1+IL-4 Mφ | IL-34+IL-4 Mφ |
|---|---|---|---|---|
| Mmp12 | 1.2395198355563393 | 4.569447294905967 | 5.951913205055021 | 5.973813020367782 |
### Chart
| Category | CSF-1 Mφ | IL-34 Mφ | CSF-1+IL-4 Mφ | IL-34+IL-4 Mφ |
|---|---|---|---|---|
| Mmp19 | 0.026306236681185417 | 0.019461745683919448 | 0.22681620167626504 | 0.29693776643591907 |
### Chart
| Category | CSF-1 Mφ | IL-34 Mφ | CSF-1+IL-4 Mφ | IL-34+IL-4 Mφ |
|---|---|---|---|---|
| Mmp13 | 0.007546056882656731 | 0.009717393073230001 | 0.15189810991645897 | 0.25614500360335224 |
### Chart
| Category | CSF-1 Mφ | IL-34 Mφ | CSF-1+IL-4 Mφ | IL-34+IL-4 Mφ |
|---|---|---|---|---|
| Retnla | 4.9053649043442454e-06 | 1.9628172645371506e-06 | 1.4403141045148589 | 1.5566382255683817 |
### Chart
| Category | CSF-1 Mφ | IL-34 Mφ | CSF-1+IL-4 Mφ | IL-34+IL-4 Mφ |
|---|---|---|---|---|
| Arg1 | 0.0002468234538433537 | 0.0002303698354055886 | 0.9023856614259927 | 0.42759379639730777 |
### Chart
| Category | CSF-1 Mφ | IL-34 Mφ | CSF-1+IL-4 Mφ | IL-34+IL-4 Mφ |
|---|---|---|---|---|
| Il1b | 0.0050737004776017095 | 0.0015659155880279583 | 0.0011359734937365338 | 0.0007459390475570758 |2-⊿Ct value
Immunosuppressive
molecules
### Chart
| Category | CSF-1 Mφ | IL-34 Mφ | CSF-1+IL-4 Mφ | IL-34+IL-4 Mφ |
|---|---|---|---|---|
| PD-L1 | 0.008527508639478038 | 0.004324199743304199 | 0.01666606349208202 | 0.014755681031679695 |
### Chart
| Category | CSF-1 Mφ | IL-34 Mφ | CSF-1+IL-4 Mφ | IL-34+IL-4 Mφ |
|---|---|---|---|---|
| PD-L2 | 6.233453620169572e-05 | 6.7381765313357095e-06 | 0.012403117719804537 | 0.007306194210256851 |■ CSF-1 Mf
■ IL-34 Mf
■ CSF-1+IL-4 Mf
■ IL-34+IL-4 Mf
B Cell surface molecular expressions (FACS)
Mouse
CSF-1+IL-4 Mf
Cell counts
F4/80
CD206
I-A/I-E
H-2 Db/Kb
PD-L1
PD-L2
C T cell inhibition assay
T cell proliferation without co-culture
CD8
CD4
Cell counts
Cocultured with CSF-1+IL-4 Mf
CFSE
D Cell surface molecular expressions (FACS)
Human
CSF-1 +IL-4 Mf
Cell counts
CD206
PD-L1
PD-L2
HLA-DR
HLA-ABC
